# HIV-1 Vpr Triggers Natural Killer Cell–Mediated Lysis of Infected Cells through Activation of the ATR-Mediated DNA Damage Response

**DOI:** 10.1371/journal.ppat.1000613

**Published:** 2009-10-02

**Authors:** Jeffrey Ward, Zachary Davis, Jason DeHart, Erik Zimmerman, Alberto Bosque, Enrico Brunetta, Domenico Mavilio, Vicente Planelles, Edward Barker

**Affiliations:** 1 Department of Microbiology and Immunology, State University of New York, Upstate Medical University, Syracuse, New York, United States of America; 2 Department of Immunology and Microbiology, Rush University Medical Center, Chicago, Illinois, United States of America; 3 Department of Pathology, University of Utah School of Medicine, Salt Lake City, Utah, United States of America; 4 Laboratorio di Immunologia Sperimentale, IRCCS, Istituto Clinico Humanitas, Rozzano (Milano), Italy; Northwestern University, United States of America

## Abstract

Natural killer (NK) cells are stimulated by ligands on virus-infected cells. We have recently demonstrated that NK cells respond to human immunodeficiency virus type-1 (HIV-1)-infected autologous T-cells, in part, through the recognition of ligands for the NK cell activating receptor NKG2D on the surface of the infected cells. Uninfected primary CD4^pos^ T-cell blasts express little, if any, NKG2D ligands. In the present study we determined the mechanism through which ligands for NKG2D are induced on HIV-1-infected cells. Our studies reveal that expression of *vpr* is necessary and sufficient to elicit the expression of NKG2D ligands in the context of HIV-1 infection. Vpr specifically induces surface expression of the unique-long 16 binding proteins (ULBP)-1 and ULBP-2, but not ULBP-3, MHC class I-related chain molecules (MIC)-A or MIC-B. In these studies we also demonstrated that Vpr increases the level of ULBP-1 and ULBP-2 mRNA in primary CD4^pos^ T-cell blasts. The presence of ULBP-1 and ULBP-2 on HIV-1 infected cells is dependent on the ability of Vpr to associate with a protein complex know as Cullin 4a (Cul4a)/damaged DNA binding protein 1 (DDB1) and Cul4a-associated factor-1(DCAF-1) E3 ubiquitin ligase (Cul4a^DCAF-1^). ULBP-1 and -2 expression by Vpr is also dependent on activation of the DNA damage sensor, ataxia telangiectasia and rad-3-related kinase (ATR). When T-cell blasts are infected with a *vpr*-deficient HIV-1, NK cells are impaired in killing the infected cells. Thus, HIV-1 Vpr actively triggers the expression of the ligands to the NK cell activation receptor.

## Author Summary

Natural killer (NK) cells are part of the innate immune response against virus infection and cancer. Recently we demonstrated that ligands for the NK cell activation receptor, NKG2D, trigger NK cell-mediated response to infected cells. These ligands are expressed on HIV-1-infected cells and not on uninfected cells. Despite the observation that NKG2D ligands are expressed on infected cells, it is unclear how HIV-1 induces their expression. In the present study, we demonstrate that HIV induces the ligands of the NKG2D receptor through the viral gene product Vpr. Vpr triggers a DNA damage response in infected cells, which in turn, increases virus production. We also demonstrate that by blocking the activity of ATR, a major component in the DNA damage response, we were able to prevent NKG2D ligand expression. When Vpr was removed from the virus genome, NK cells lost their ability to lyse the HIV-infected cells. Thus, HIV-1 actively triggers NK cells through the activity of its viral gene product, Vpr.

## Introduction

NK cells are involved in the immune response against tumor cells and virus-infected cells without the requirement for previous exposure to their targets or their products. The importance of NK cells in restraining viral infection was first shown in studies using murine models in which NK cells were depleted [1],[2],[3]. In these studies, NK cell depletion led to enhanced viral replication and cytopathology. In humans, NK cells control the severity of viral infections such as those by herpes simplex virus [4],[5], cytomegalovirus [6] and hepatitis B virus [7]. Lack of NK cells in humans or defects in NK cell function are associated with fatal disseminated herpes virus infection [8],[9],[10],[11],[12],[13].

One of the major roles of NK cells in controlling viruses is the destruction of the infected cells. Direct target cell killing by NK cells is mediated by the regulated release of granules containing perforin and granzymes [14],[15]. Perforin forms pores in the plasma membrane allowing ions and small particles into and out of the cell [16]. The granzymes most likely enter the target cell through perforin-formed channels [17] or endocytosis [16] and induce apoptosis of the infected cells. NK cells may also kill through engagement of death receptors (e.g., Fas) by ligands expressed on the NK cell surface (e.g., CD178) whose surface expression is increased upon degranulation [18]. *In vivo*, cytotoxic function is primarily mediated by the subset of NK cells that are CD56^dim^/CD16^pos^, the dominant subset in the peripheral blood of healthy individuals [19]. CD56^bright^/CD16^neg^ cells constitute a minor NK population in the blood but are the major NK cell subset in secondary lymphoid tissues. CD56^bright^ NK cells express less perforin than CD56^dim^ NK cells but higher concentrations of cytokines and as such are important in regulating immune responses.

NK cell release of cytotoxic contents is regulated by a large array of signals provided by a variety of membrane-bound, activating receptors expressed on NK cells that interact with their corresponding ligands on target cells (see [20] for a current list of all known activating receptors and their ligands). Almost all NK cell activating receptors interact with adaptor proteins containing activation domains [20]. Activation receptors can be divided into those associated with intracellular tyrosine activation motifs (ITAM)-containing adaptor molecules and those associating with adaptor molecules lacking ITAMs. ITAM-dependent receptors include: CD16 (low affinity IgG receptor), and the natural cytotoxicity receptors (NCRs; NKp30, NKp44 and NKp46) [21]. The NKG2D receptor is an example of an ITAM-independent activation receptor. NKG2D is found on almost all peripheral blood NK cells and is a member of the NKG2 receptor family. NKG2D does not associate with CD94, but forms homo-dimers on the membrane of NK cells [22],[23]. Although NKG2D specifically recognizes its ligands on target cells and is important for activation of NK cells, it does not induce signal transduction within the cells. For this purpose, NKG2D associates, on the membrane, with the DAP10 adaptor molecule. The DAP10 adaptor protein contains a YXXM motif instead of ITAM and recruits phosphatidylinositol-3- kinase and Grb-2 upon phosphorylation [20].

Our prior studies [24],[25] and those of others [26] indicate that HIV-1-infection of primary CD4^pos^ T-cells leads to the surface expression of NKG2D ligands. The ligands for human NKG2D are the MIC-A and -B and ULBP 1–4 [27],[28]. ULBPs but not MIC-A or -B were found on the surface of HIV-infected cells in our prior studies. We not only found these molecules on *in vitro* infected primary CD4^pos^ T-cell blasts but also on infected cells obtained from HIV-1-infected patients after amplification of the virus-infected cells *ex vivo* [25]. In addition, these studies, which used primary CD4^pos^ T-cells infected with HIV-1 as targets for autologous NK cells in cytotoxicity assays, revealed that NK cells can respond to the HIV-1-infected cells in an NKG2D-dependent manner [24],[25].

A recent study by Gasser *et al.* demonstrated, that DNA damage induced expression of a plethora of NKG2D ligands [29]. The up-regulation of NKG2D ligands occurred after the activation of the DNA damage recognition enzymes ataxia telangiectasia mutated (ATM) and ATR [29]. The HIV-1 Vpr protein is a potent activator of ATR, and induces infected cells to arrest in the G_2_ phase of the cell cycle, [30],[31] and increases viral transcription from the HIV-1 long-terminal repeat [32],[33],[34]. Our previous studies have demonstrated that the ability of Vpr to induce cell cycle arrest in G_2_ is dependent on activation of ATR but not ATM [30],[35]. Therefore, we hypothesized that HIV-1 Vpr is responsible for inducing the expression of NKG2D ligands during infection through activation of ATR. We further hypothesized that Vpr-mediated up-regulation of NKG2D ligands would lead to NK cell activation. If these hypotheses are correct, this would indicate that the DNA damage signaling by Vpr has consequences that may potentially be detrimental to the virus because they enhance immune surveillance by NK cells.

## Results

### Vpr induces expression of the NKG2D ligands, ULBP-1 and ULBP-2

Initially, we determined the role of Vpr in the up-regulation of NKG2D ligands on primary CD4^pos^ T-cells. To eliminate possible differences in replication kinetics due to the presence or absence of Vpr [32],[33], we used a defective HIV-1 construct, DHIV, that has a deletion in the *env* gene. We then provided the VSV-G glycoprotein *in trans*, to form pseudotyped virions [36],[37]. In humans, six different NKG2D ligands have been reported to date. In order to be able to detect global changes in the expression of all six NKG2D ligands, we utilized a recombinant soluble NKG2D receptor that binds to all of them. We stained the cells with viability dyes and evaluated only the viable infected population for expression of NKG2D ligands (Figure S1).

**Figure 1:**
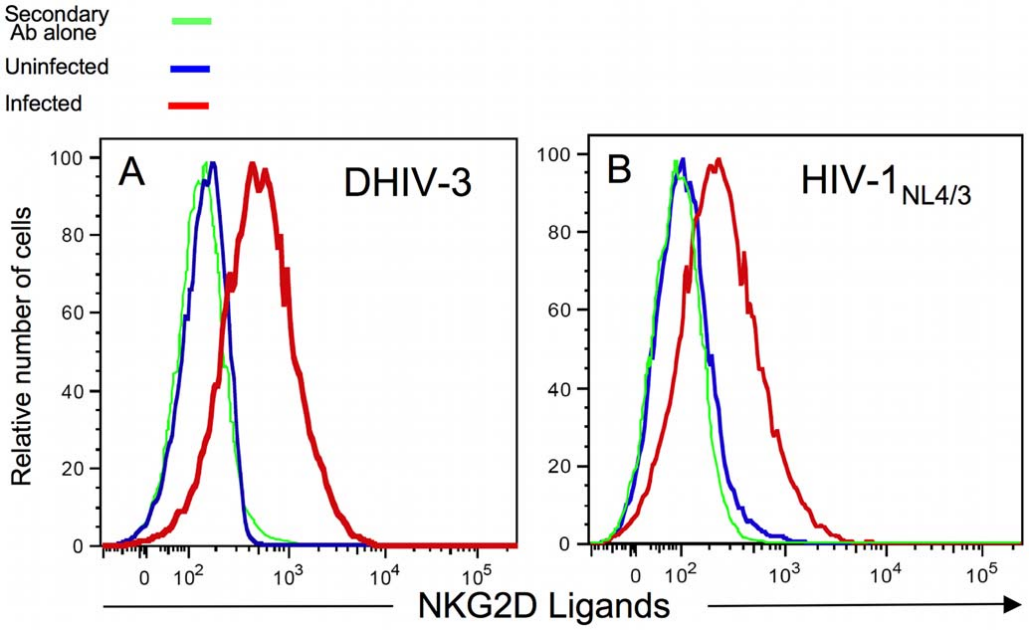
Infected primary CD4^pos^ T-cells express NKG2D ligands. DHIV (A) and HIV-1_NL4/3_ (B) -infected primary T-cell blasts and uninfected CD4^pos^ T-cells were surface stained with fluorochrome-conjugated anti-CD4 Ab and a fusion protein of human NKG2D and the Fc portion of human IgG1 along fluorochrome-conjugated goat anti-human IgG1. All cells were stained intracellularly for HIV-1 p24 antigen (Ag). Histograms were derived following acquisition on a flow cytometer of either 10^4^ viable cells [(for uninfected cells) blue line] or 10^4^ viable CD4^neg^ cells and HIV-1 p24 Ag^pos^ infected cells (red line). As controls (green line), cells were stained with secondary Ab alone. This figure is representative of five separate experiments.

We tested the binding of soluble NKG2D as a measure of ligand expression in PBMC from five individuals, in the presence or absence of *in vitro* DHIV infection. As seen in Figure 1A, CD4^pos^ T-cells infected with DHIV wild type (WT) expressed NKG2D ligands [MFI = 687 for infected cells (HIV-1 p24 Ag^pos^/CD4^neg^) compared with MFI = 159 for uninfected control]. Experiments were performed in parallel for a total of 5 donors and the statistical difference in MFI between the infected and uninfected groups in five individuals was p<0.01 based on the Student's t-test. Uninfected cells did not detectably express NKG2D ligands (MFI = 152 for uninfected cells compared with MFI = 159 for secondary Ab staining). Within the infected population, only the HIV-1 p24 Ag^pos^ cells, but not the p24^neg^ in the same culture, expressed NKG2D ligands (see Figures 1 and S2). Therefore, we conclude that NKG2D is not induced on uninfected, bystander cells. DHIV does not encode the envelope glycoprotein; however NKG2D ligands are also induced on envelope-expressing HIV-1_NL4/3_-infected cells (Figure 1B). DHIV is derived from HIV-1_NL4/3._ Thus, DHIV-infected cells express NKG2D ligands on their cell surface.

**Figure 2 ppat-1000613-g002:**
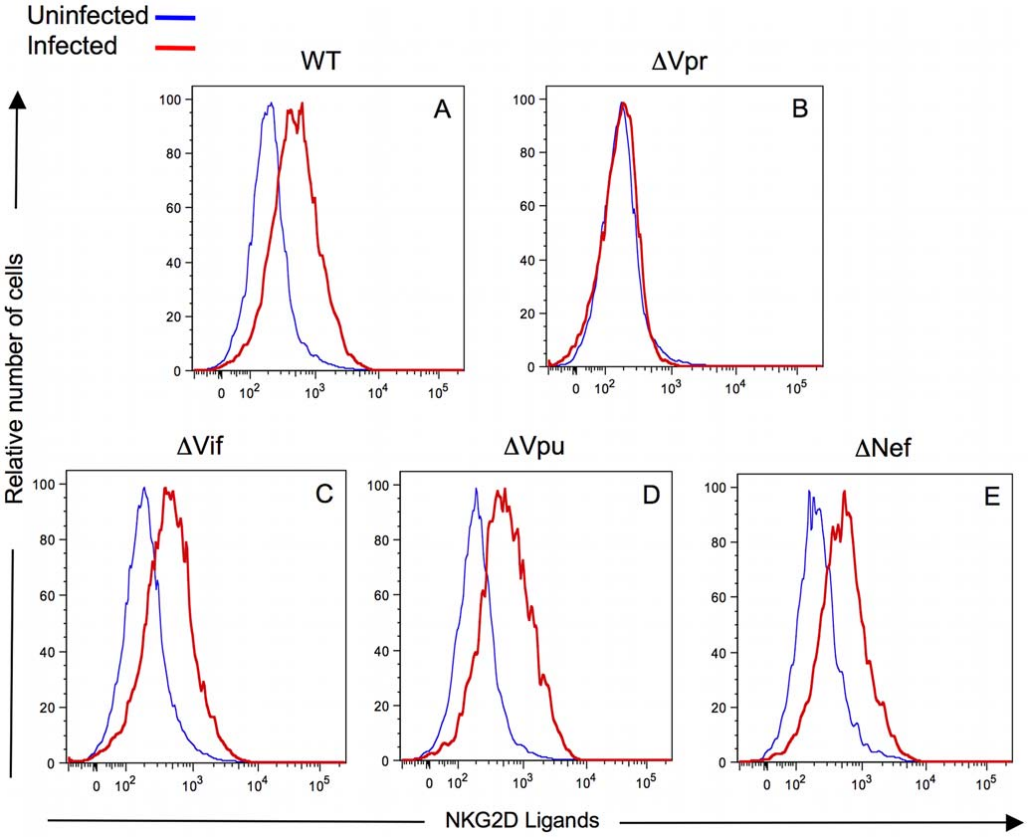
Expression of NKG2D ligands on CD4^pos^ T-cells following infection with viruses unable to express Vpr, Vif, Vpu or Nef. Infected primary T-cell blasts [(A) Wild-type, (B) ΔVpr, (C) ΔVif, (D) ΔVpu and (E) ΔNef] and uninfected CD4^pos^ T-cells were surface stained with fluorochrome-conjugated anti-CD4 Ab and a fusion protein of human NKG2D and the Fc portion of human IgG1 along with fluorochrome-conjugated goat anti-human IgG1. All cells were stained intracellularly for HIV-1 p24 Ag. Histograms were derived following acquisition on a flow cytometer of either 10^4^ viable CD4^pos^ cells [(for uninfected cells) blue line] or 10^4^ viable CD4^neg^ and HIV-1 p24 Ag^pos^ infected cells (red line). The figure is representative data from three separate experiments.

We then sought to determine whether Vpr was responsible for the expression of NKG2D ligands. We generated DHIV containing a truncation in Vpr. As controls, mutants of DHIV unable to express Vif, Vpu or Nef were also generated. Figures 2 and S2 illustrate that the DHIV-ΔVpr infected cells failed to induce NKG2D ligand expression (Figure 2B; MFI = 188 for HIV-1 p24 Ag^pos^/CD4^neg^ infected cells and MFI = 216 for HIV-1 p24 Ag^neg^/CD4^pos^ infected cells). The MFI for DHIV-WT infected cells was statistically different (p<0.01) compared to the MFI for DHIV-ΔVpr infected cells. We compared expression of NKG2D ligands on CD4^pos^ T-cells infected with DHIV-ΔVif (Figure 2C; MFI = 600 for HIV-1 p24 Ag^pos^/CD4^neg^ infected cells and MFI = 260 for HIV-1 p24 Ag^neg^/CD4^pos^ infected cells), DHIV-ΔVpu (Figure 2D; MFI = 777 for HIV-1 p24 Ag^pos^/CD4^neg^ infected cells and MFI = 247 for HIV-1 p24 Ag^neg^/CD4^pos^ infected cells), and DHIV-ΔNef (Figure 2E; MFI = 698 for HIV-1 p24 Ag^pos^/CD4^neg^ infected cells and MFI = 303 for HIV-1 p24 Ag^neg^/CD4^pos^ infected cells). Therefore, expression of NKG2D ligands induced by ΔVif, ΔVpu and ΔNef DHIV was comparable that induced by WT DHIV (Figure 2A). Thus, we conclude that Vpr is required for HIV-1-mediated up-regulation of NKG2D ligands on the surface of infected cells.

**Figure 3:**
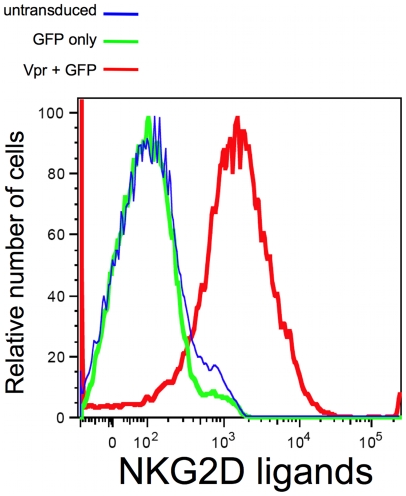
Vpr alone is capable of inducing NKG2D ligands surface expression. Primary CD4^pos^ T-cell blasts were transduced with a lentivirus vector (pPR-Vip) coexpressing Vpr and GFP (red line). As a control the same cells were infected with a lentivirus vector expressing GFP only (green line). Following transduction cells were stained with a fusion protein of human NKG2D and the Fc portion of human IgG1 along with fluorochrome-conjugated goat anti-human IgG1. Histograms were derived following acquisition on a flow cytometer of either 10^4^ viable cells [(for untransduced cells) blue line] or 10^4^ viable GFP^pos^ cells (green and red line). The figure is representative data from two separate experiments.

To determine whether Vpr expression is sufficient to induce NKG2D ligands, we resorted to a lentiviral vector that encodes HIV-1 Vpr and GFP but no other viral gene (pPR-VIP). As a control, we used a similar lentiviral vector encoding only GFP. We observed that Vpr alone, but not the control lentiviral vector, was able to induce NKG2D ligands on CD4^pos^ T-cells (Figure 3). Therefore, Vpr is sufficient for HIV-1 to induce NKG2D ligands on the infected cell surface.

**Figure 4:**
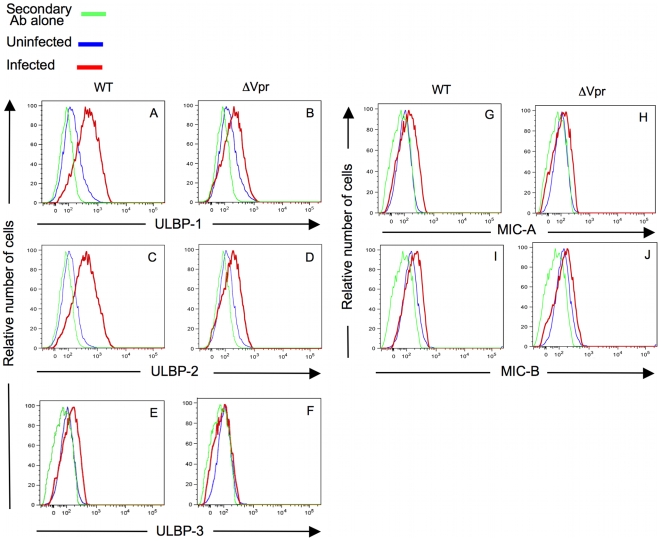
Vpr is more likely to induce surface expression of ULBP-1 and ULBP-2 than ULBP-3, MIC-A or MIC-B on infected cells. Primary CD4^pos^ T-cell blasts were infected with wild-type (WT) HIV-1. As a control, the same cells were infected with HIV-1 that was deficient in expression of Vpr (ΔVpr). As an additional control we evaluated uninfected CD4^pos^ T-cells for expression of individual NKG2D ligands (blue lines). Following infection, cells were surface stained using fluorochrome-conjugated mAb specific for: CD4 (A-J), ULBP-1 (A–B), ULBP-2 (C–D), ULBP-3 (E–F), MIC-A (G–H) or MIC-B (I–J). The cells were then intracellularly stained for HIV-1 p24 antigen. Histograms were derived following acquisition of 10^4^ viable uninfected CD4^pos^ cells (blue line) or 10^4^ viable CD4^neg^ and HIV-1 p24 Ag^pos^ infected cells (red line). Green line represents the histogram of staining controls (isotype controls). The figure is representative data from two separate experiments.

The studies illustrated in Figures 1–3 utilized a soluble NKG2D construct that is unable to distinguish between the various ligands. Hence, we wished to determine which of the NKG2D ligands were specifically induced by Vpr. For this purpose, we compared the expression of ULBP-1, ULBP-2, ULBP-3, MIC-A and MIC-B on DHIV-infected cells. In the studies shown in Figure 4, we demonstrate that WT virus induced ULBP-1 (Figure 4A; MFI = 483 for WT virus-infected cells compared with MFI = 167 for uninfected cells) and ULBP-2 (Figure 4C; MFI = 539 for WT virus-infected cells compared with MFI = 131 for uninfected cells). In contrast, little or no induction of ULBP-3 (Figure 4E; MFI = 150 for WT virus-infected cells compared with MFI = 134 for uninfected cells), MIC-A (Figure 4G; MFI = 187 for WT virus-infected cells compared with MFI = 106 for uninfected cells) or MIC-B (Figure 4I; MFI = 99.8 for WT virus-infected cells compared with MFI = 90.5 for uninfected cells) was detected.

DHIV-ΔVpr induced considerably lower levels of ULBP-1 (Figure 4B; MFI = 220 for ΔVpr virus-infected cells compared with MFI = 485 for WT virus-infected cells) and ULBP-2 (Figure 4D; MFI = 179 for ΔVpr virus-infected cells compared with MFI = 539 for WT virus-infected cells) than WT virus. Thus, HIV-1 primarily induces the expression of ULBP-1 and ULBP-2 through Vpr.

### Induction of ULBP-1 and ULBP-2 by Vpr occurs at the level of gene expression

**Figure 5:**
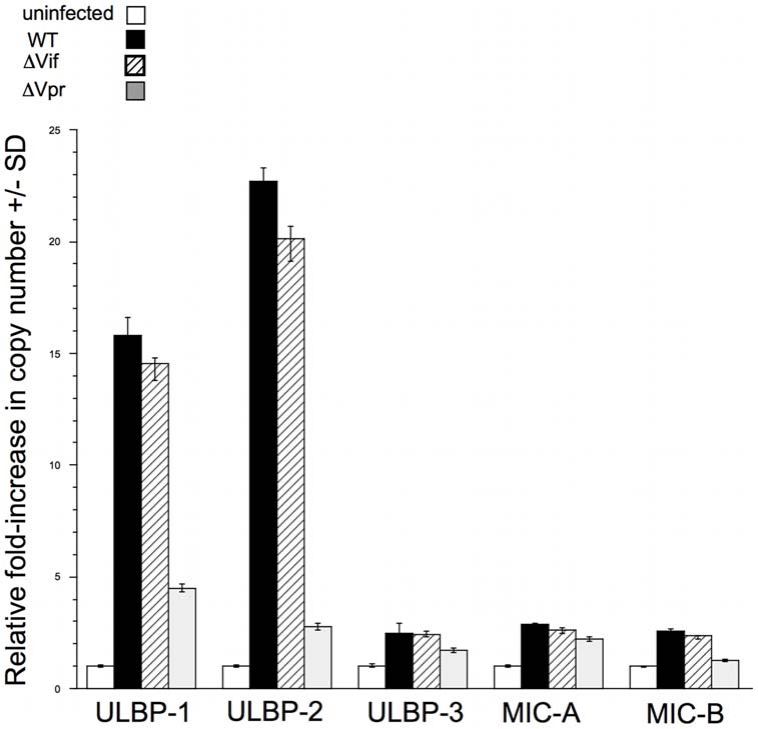
Vpr is involved in upregulating the expression of ULBP-1 and ULBP-2 mRNA. RNA was extracted from HIV-infected CD4^pos^ T-cell blasts and uninfected cells and reverse transcribed. Relative copy numbers of NKG2D ligands were determined by real-time PCR using NKG2D-ligand specific primer pairs and normalized relative to GAPDH expression. The relative increase in copy numbers was calculated as described in Materials and Methods. The relative copy numbers for the indicated primer pair in uninfected cells was set to 1. Error bars indicate the standard deviations of the mean of samples in triplicate.

Next we asked whether induction of NKG2D ligands on infected cells was accomplished through increased steady-state levels of ULBP-1 and ULBP-2 mRNA. We infected CD4^pos^ T-cells with DHIV and select mutants, and then measured the mRNA levels of ULBP-1, ULBP-2, ULBP-3, MIC-A and MIC-B relative to the level of GADPH mRNA (Figure 5). We found that both ULBP-1 and ULBP-2 mRNA levels were 15-fold higher in WT virus-infected cells compared with ULBP-1 and ULBP-2 gene products in uninfected cells. In comparison to WT DHIV-infected cells, DHIV-ΔVif-infected cells showed little or no changes in ULBP-1 and ULBP-2 mRNA levels. In addition, in DHIV-ΔVpr-infected cells, the levels of ULBP-1 and -2 mRNA were 3 to 10-fold lower compared with those in WT DHIV-infected cells (Figure 5). Infection with WT DHIV only induced a 1.5-fold increase in the expression ULBP-3, MIC-A and MIC-B. Thus, among the five NKG2D ligands we evaluated, Vpr is responsible for up-regulating the levels of ULBP-1 and ULBP-2 mRNA in HIV-1-infected cells.

### Expression of NKG2D ligands requires Vpr's ability to interact with the Cullin 4a^DCAF-1^ E3 ubiquitin ligase and to activate ATR

Vpr induces G_2_ arrest of infected cells through interaction with a Cul4a-based ubiquitin ligase that also contains the adaptor, DDB1, and the substrate receptor, DCAF1 (reviewed in [38]). Despite the fact that the degradation target for this ubiquitin ligase is unknown, the consequences of the Vpr-E3 complex are well documented. The main result is the activation of the ATR kinase [39] that, in turn, leads to G_2_ arrest [35]. Therefore, we wished to determine whether Vpr recruitment of the Cullin-4a-based E3 ubiquitin ligase complex is required for ULBP-1 and -2 expression.

**Figure 6:**
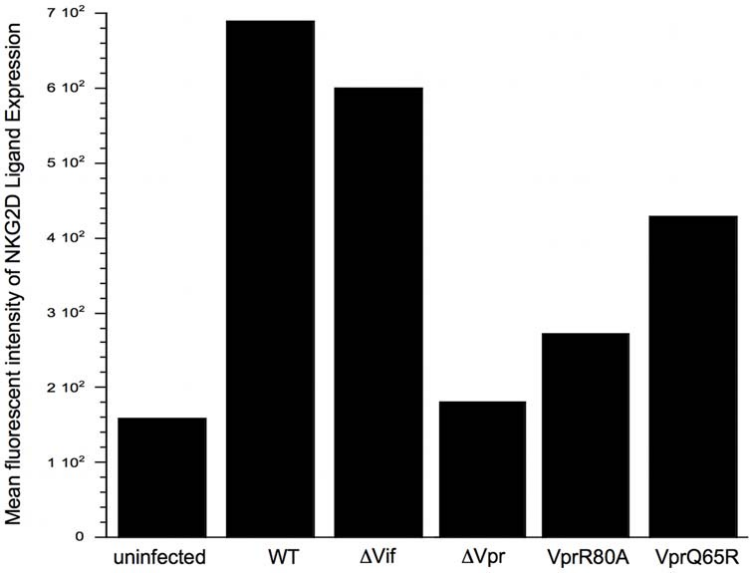
Regions of Vpr that are involved in the ability of Vpr to induce arrest in the cell cycle at the G_2_ phase are also important in NKG2D ligand expression. Primary T-cell blasts infected with wild type HIV-1 and HIV-1 with various mutations in Vpr were surface stained with fluorochrome-conjugated anti-CD4 Ab and a fusion protein of human NKG2D and the Fc portion of human IgG1 along with fluorochrome-conjugated goat anti-human IgG1. Uninfected CD4^pos^ T-cells were surface stained in a similar fashion. All cells were stained intracellularly for HIV-1 p24 Ag. The mean fluorescent intensity of NKG2D ligand staining was obtained from collection of 10^4^ CD4^neg^ p24^pos^ cells for all infected cells, and of the 10^4^ CD4^pos^ p24^neg^ cells for the uninfected control. This figure is a representative of three separate experiments.

We first tested whether domains in Vpr that are involved in recruiting or activating the Cullin-4a-based E3 ubiquitin ligase were required for induction of ULBP-1 and ULBP-2. The Vpr Q65R and Vpr R80A mutations have been previously shown to abate the ability of Vpr to induce cell cycle arrest [39],[40]. Vpr Q65R is unable to bind to DCAF1; the exact defect induced by R80A mutation is unknown, and it has been proposed that R80A abates the interaction between Vpr and the ubiquitination target for the E3/Vpr complex [39],[40]. For this purpose, we generated DHIV mutants with the above substitutions in Vpr. As shown in Figure 6, expression of NKG2D ligands was diminished by either substitution, although Vpr(R80A) had a more dramatic effect than Vpr(Q65R) (18 percent of wild-type Vpr and 48 percent of wild-type Vpr, respectively). The cell cycle profiles were examined in infected cells to visualize functional deficits in these mutants. When Vpr R80A or Vpr Q65R, were expressed neither mutant virus could induce G_2_ arrest as effectively as WT virus (Figure S3).

**Figure 7:**
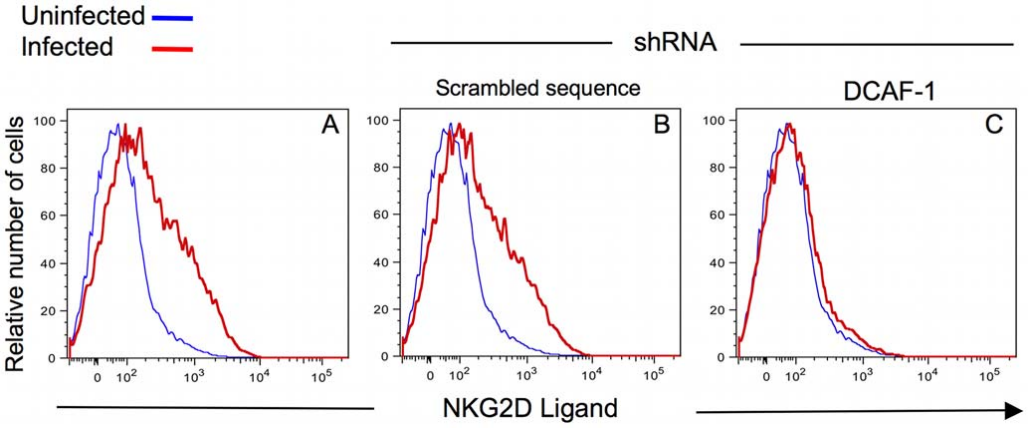
The DCAF1 subunit of Cul4a E3 ubiquitin ligase is involved in the ability of HIV-1 to induce surface expression of NKG2D ligands. Primary CD4^pos^ T-cell blasts were transduced with shRNA of a DCAF1-specific sequence (C) or an shRNA with a scrambled sequence (B). Untransduced cells were used as a negative control (A). The cells were then infected and stained with anti-CD4 Ab and a fusion protein of human NKG2D and the Fc portion of human IgG1 along with fluorochrome-conjugated goat anti-human IgG1. Transduced cells were detected by GFP expression. The figure is representative data from two separate experiments.

To more directly assess the requirement of the Cullin-4a-based E3 ubiquitin ligase in inducing NKG2D ligand expression, we resorted to RNA interference-mediated depletion of DCAF1 (Figure 7). Results shown in Figure S4 demonstrate that infection with HIV-1 and indicated mutant viruses had no effect on the levels of DCAF1 protein. We generated a lentivirus vector, based on FG12 [41], expressing short hairpin RNAs (shRNAs) specific for DCAF1 [42]. Knockdown of DCAF1 expression completely abated the ability of WT DHIV to up-regulate NKG2D ligand expression (Figure 7C) compared with the same infected cells expressing a control (scrambled sequence) shRNA (Figure 7B) or untransduced cells (Figure 7A). Thus, NKG2D ligand expression on HIV-1-infected cells is dependent on the Cul4a^DCAF-1^ ubiquitin ligase complex.

**Figure 8:**
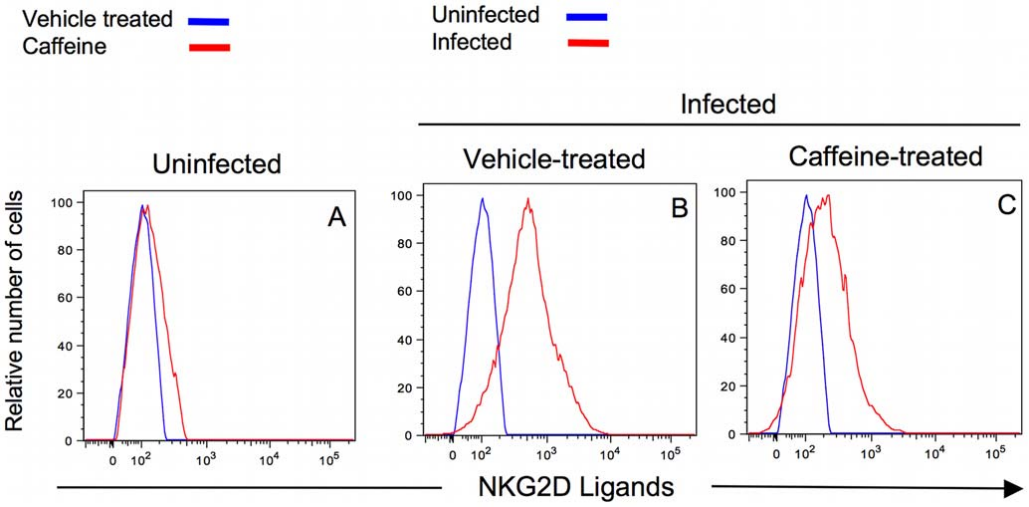
Inhibition of ATR activity relieves Vpr-induced NKG2D-ligand expression. Primary CD4^pos^ T-cells were infected in the presence (C) or absence (B) of the ATR inhibitor, caffeine. As controls CD4^pos^ T-cells were not infected (A) but treated with caffeine or vehicle. Following exposure to 4 mM caffeine and HIV-1 infection, NKG2D ligand expression on the surface of infected cells was measured by staining with a fusion protein of human NKG2D and the Fc portion of human IgG1 along with fluorochrome-conjugated goat anti-human IgG1. The histograms are gated for either 10^4^ viable CD4^pos^/HIV-1 p24^neg^ cells (blue) or on 10^4^ viable CD4^neg^ HIV-1 p24^pos^ cells (red). This figure is representative of three separate experiments.

Manipulation of the Cul4a^DCAF-1^ complex by Vpr results in ATR activation and G_2_ arrest [37]. It is formally possible that up-regulation of ULBP-1 and -2 is also a consequence of manipulation of Cul4a^DCAF-1^, but independent of ATR activation. Alternatively, ATR activation may be required for ULBP up-regulation, as was suggested by Gasser et al [29]. To differentiate between these two possibilities, we treated DHIV-infected cells with caffeine, a known inhibitor of ATR and its related kinase, ATM [30],[35],[43]. As shown in Figure S5, caffeine, at a concentration of 4 mM, eliminated the ability of HIV-1 to induce G_2_ arrest from a ratio of G_2_+M/G_1_ = 7.67 to ratio of G_2_+M/G_1_ = 0.53. Caffeine (4 mM) treatment of HIV-1-infected cells dampened NKG2D ligand expression (Figure 8; MFI = 277 for caffeine-treated virus-infected cells compared to MFI = 763 for vehicle-treated virus-infected cells). Caffeine had little effect on NKG2D ligand expression on uninfected cells (Figure 8; MFI = 148 for caffeine-treated uninfected cells compared to MFI = 103 for vehicle-treated uninfected cells). Thus, caffeine inhibition of ULBP up-regulation suggests that ATR is a required upstream mediator of NKG2D ligand expression.

**Figure 9:**
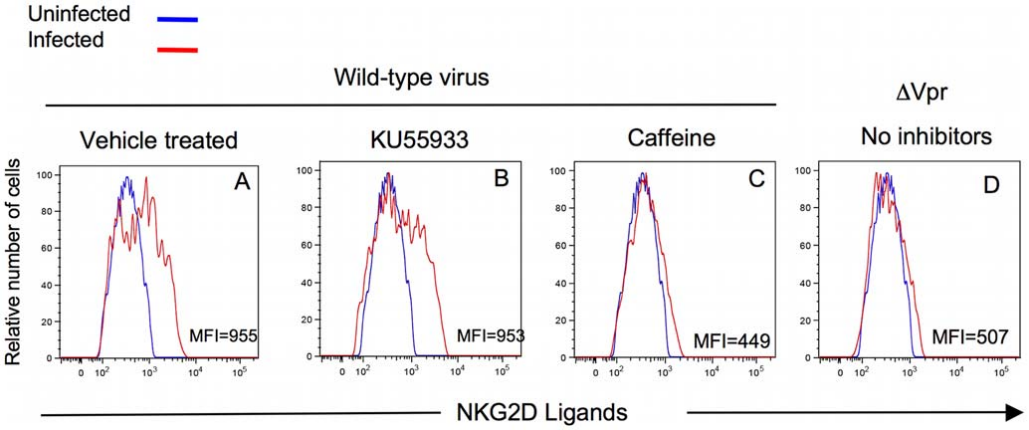
Inhibition of ATM does not affect NKG2D-ligand expression on infected cells. Primary CD4^pos^ T-cells were infected in the presence (B) of the ATM-specific inhibitor KU55933 (10 µM). As a positive control infected cells were treated with 4 mM caffeine (C). Negative controls included vehicle-treated infected cells (A) or CD4^pos^ T-cells infected with HIV-1 lacking Vpr [(ΔVpr) D]. Following exposure to inhibitors and HIV-1 infection, NKG2D ligand expression on the surface of infected cells was measured by staining with a fusion protein of human NKG2D and the Fc portion of human IgG1 along with fluorochrome-conjugated goat anti-human IgG1. The histograms are gated for either 10^4^ viable CD4^pos^/HIV-1 p24^neg^ cells (blue) or on 10^4^ viable CD4^neg^ HIV-1 p24^pos^ cells (red). MFI = mean fluorescent intensity. This figure is representative of three separate experiments.

Since caffeine inhibits both ATR and ATM [43] it is formally possible that the effect of HIV Vpr on ULBP up-regulation is dependent on both ATR and ATM. Thus, to determine any role that ATM may play in the induction of NKG2D ligands by HIV, we evaluated the effect of KU55933, an ATM-specific inhibitor on NKG2D ligand expression [44]. Figure 9 shows that treatment of infected cells with 10 µM KU55933 had little effect on NKG2D ligand expression (MFI = 953) relative to infected cells treated with vehicle alone (MFI = 955). In contrast, KU55933 did affect the level of NKG2D ligands expressed on primary CD4^pos^ T-cells following treatment with aphidicolin, a DNA polymerase α inhibitor which induces replication stress (MFI = 432) relative to vehicle-treated cells (MFI = 792) (Figure S6). Unlike KU55933, caffeine prevented NKG2D ligand expression on both HIV-1-infected and aphidicolin treated cells (see Figures 9 and S6). Thus, Vpr induces NKG2D ligand through activation of ATR but not ATM.

### Up-regulation of ULBP 1 and 2 by Vpr contributes to killing by NK cells

**Figure 10:**
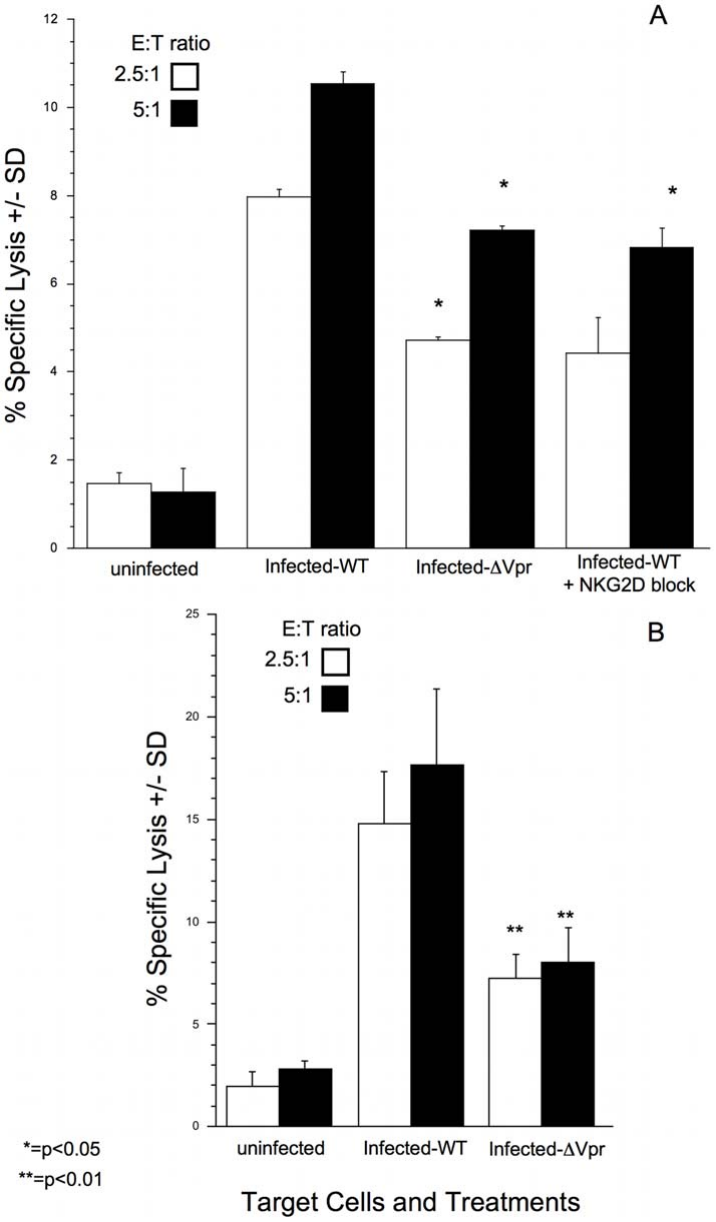
Vpr is involved in triggering NK cells to kill the infected cell. Primary CD4^pos^ T-cell blasts were infected with HIV-1 that was deficient in expression of Vpr (ΔVpr) (A&B). As a control, the same cells were infected with wild-type (WT) HIV-1 (A&B). Following infection the infected cells were isolated, labeled with ^51^Cr and mixed with autologous NK cells at 2.5∶1 (open bars) and 5∶1 (closed) effector cell to target cell ratios. Each group was done in triplicate. Prior to the lytic assay some of the NK cells were exposed to blocking antibodies to NKG2D (A). At the end of the incubation period culture fluids were harvested and analyzed for the presence of ^51^Cr. Percent specific lysis was determined as described in the Materials and Methods section. Error bars represent standard deviation of the mean. The figure is representative of two separate experiments. *p<0.05, **p<0.01 by the Student's t-test.

Our studies, thus far, demonstrate that HIV-1 induces the expression of ULBP-1 and ULBP-2 through Vpr. However, whether Vpr mediated-induction of ULBP-1 and ULBP-2 on infected cells constitutes a signal that will trigger NK cell lysis remains to be determined. To address this, we compared the ability of primary NK cells to lyse autologous T-cell blasts when infected with either WT or ΔVpr viruses. As a control, we blocked NKG2D on NK cells prior to exposure to target cells. If NKG2D interaction with its ligands on infected cells had any role in triggering NK cell lysis, then blocking NKG2D would abate the effect. As shown in Figure 10, cells infected with WT virus are sensitive to NK cell lysis, when compared with uninfected cells. Infection with DHIV-ΔVpr led to a reduced level of NK-mediated lysis (Figures 10 and S7). Although the reduction was not complete, it is noteworthy that deletion of Vpr had a similar effect as the blockade of NKG2D (Figure 10A). These observations, taken together, indicate that up-regulation of NKG2D ligands by Vpr is responsible for a fraction of the observed lytic activity by NK cells. Thus, we conclude that through its known abilities to interact with the Cullin 4a-based E3 ubiquitin ligase and activate ATR, Vpr induces expression of ULBP-1 and -2 and this, in turn, constitutes a signal that triggers NK cell lysis of infected cells.

## Discussion

Our previous studies demonstrated that primary T-cell blasts infected both *in vitro* and *in vivo* with HIV expressed, on their cell surface, ligands for NKG2D [24],[25]. Here, we demonstrate that HIV-1 Vpr selectively induces expression of ULBP-1 and ULBP-2 gene products (Figures 4 and 5). ULBP-3, MIC-A and MIC-B were hardly, if at all, up-regulated. We did not observe ULBP-4 on HIV-infected cells using a recently generated anti-ULBP-4 monoclonal antibody (a gift from Dr. John Trowsdale, University of Cambridge, Cambridge, England) in a separate experiment (data not shown).

DNA damage leads to the expression of NKG2D ligands [29]; our observations here indicate that Vpr, through activation of the DNA damage pathway, induces similar effects as those reported by Gasser [29]. This ultimately leads to expression of ULBP-1 and -2 on the cell surface. Despite the potential abilities of both ATM and ATR, following DNA damage, to induce NKG2D ligands [29], only ATR in HIV-1 infected cells appears to trigger the expression of NKG2D ligands. This is consistent with the previously reported specificity of Vpr for ATR and not for ATM [30],[31]. We now show that Vpr enhances NKG2D ligand expression on infected cells and that ATR is required for this novel activity of Vpr (see Figures 8 and 9).

The available literature on whether retroviruses activate DNA damage responses upon integration remains controversial. Several publications propose that HIV (and other retroviruses) do not cause DNA damage signaling when they integrate [45],[46] while others have shown the participation and/or requirement of checkpoint proteins and DNA damage sensors [47],[48]. HIV-1 Vpr activates the DNA damage sensor, ATR, but importantly, Vpr does not do so upon integration, but only after *de novo* expression in the infected cells, well beyond the integration step [46]. Furthermore, we found no evidence that the presence of Vpr in virions early in infection enhanced the efficiency of viral integration to any degree [46]. Accordingly, Vpr has been shown to exert identical effects on the cell cycle when expressed via systems devoid of viral integration (such as adenoviral vectors, plasmid transfection, and tetracycline-inducible Vpr cell lines [49],[50],[51]).

Recent studies from other investigators indicate that soluble Vpr has an impact on NK cell activity [52]. Although we did not directly assess the role of soluble Vpr, our observations produced no evidence that soluble Vpr released by HIV-infected cells would induce NKG2D ligands on uninfected cells. We base this notion on the fact that uninfected (HIV-1 p24^neg^) cells within infected cultures do not express NKG2D ligands on their surface. Only HIV-1 p24^pos^ cells express NKG2D ligands (see Figures 1 and S2). Hence, we propose that Vpr must be expressed within the infected cells in order to induce NKG2D ligand expression.

HIV-1 accessory gene products other than Vpr appear to have no effect on the expression of NKG2D ligands. Our observations here are in contrast to those of Cerboni *et al.* who showed that HIV-1 Nef down-regulates ULBPs from the cell surface of Jurkat T-cells [26]. The contradictory observations made in our studies may suggest that there are differences in the ability of Nef to modulate NKG2D ligands between activated primary T-cells and transformed T-cell lines. The ΔNef virus we used in our study does not express Nef even though it expresses Vpr (Figure S8B).

The Cul4a^DCAF-1^ ubiquitin ligase contributes to the ability of Vpr to arrest HIV-1-infected cells in G_2_ phase of the cell cycle [38],[39]; in our current studies we showed that this ligase is important in Vpr-induced NKG2D ligand expression as well. E3 ubiquitin ligases transfer ubiquitin molecules to specific substrates. Poly-ubiquitination can mark substrates for degradation by the proteasome. We attempted to test the role of proteasomal degradation in induction of NKG2D ligands. Unfortunately, proteasome inhibitors were highly toxic to primary T-cells and, therefore, the possible role of proteasomal activity in degrading NKG2D ligands is unknown at this time (data not shown).

Our expectation was that induction of cell-surface expression of ULBP-1 and ULBP-2, would trigger NK cells to kill infected cells. This indeed is what we found, since NK cells have a decreased ability to lyse virus-infected cells lacking Vpr. Moreover, the reduction in killing activity of the ΔVpr virus was strikingly similar to that obtained against WT virus-infected cells in which we masked the NKG2D receptor. We have reported, in previous published studies, that variations exist between HIV-infected donors in the capacity of their NK cells to kill autologous HIV-infected cells [25]. We observed killing from as little as 4% and as high as 20% in 8 donors using an E∶T ratio of 10∶1 [25]. Importantly, The level to which NK cells lysed HIV-infected targets directly correlated with the extent to which ULBP2 was expressed on the surface of the infected cells [25]. Those observations provide indirect support to our model that Vpr, through enhancing ULBP expression, may prime cells for lysis by NK.

NK cell function in HIV-infected individuals is similar to that of uninfected individuals unless the patient is highly viremic [53]. These viremic patients typically possess a dysfunctional NK cell subpopulation lacking CD56 but retaining CD16 [54]. Despite the presence of dysfunctional NK cells in viremic individuals, there are subpopulations of functional NK cells in these patients [53] with residual ability to lyse autologous HIV-infected cells [25]. It has been shown that this residual activity required recognition of NKG2D ligands on the infected cell surface [25].

Based on our studies, the expected consequence of Vpr's induction of ULBP-1 and -2 would be that HIV-1-infected cells would become sensitive to killing by NK cells. This scenario would clearly be detrimental to virus replication. A logical explanation for this paradox is that up-regulation of ULBPs by Vpr is a downstream consequence of its biological activity (e.g., Vpr induces LTR transactivation in the G_2_ phase of the cell cycle [32]). Therefore, we hypothesize that other unknown cellular factors may compensate for the activity of Vpr to collectively modulate the sensitivity of HIV-infected cells to lysis by NK cells.

It is difficult to reconcile how HIV-1 could persist if it increases the likelihood of being destroyed by NK cells through induction of ULBP-1 and ULBP-2 (Figure 4) by Vpr on the cell surface and down modulating ligands (i.e., HLA-A and –B) to NK cell inhibitory receptors by Nef [55]. Part of the answer may lie in the fact that Nef does not down modulate HLA-C and HLA–E [55],[56]. We have published previously [56] that NK cells lacking HLA-C and –E inhibitory receptors kill infected cells by 5-20-fold over unsorted NK cells. Blocking HLA-C and –E on HIV-infected cells from interacting with inhibitory receptors on NK cells enhances NK cells' ability to kill HIV-infected cells [56]. Overall these findings indicate that HLA-C and –E on the infected cells prevent NK cells from killing the infected cells because inhibitory receptors are engaged on NK cells. However, our previous study also indicated that a sufficient number of NK cells lack HLA-C and HLA–E inhibitory receptors and thus would not be inhibited by these MHC class I molecules [55],[56].

The presence of ULBP-1, ULBP-2 on the infected cell surface would allow the subpopulation of NK cells not regulated by HLA-C and HLA–E to kill the HIV-1-infected cells. However, NK cells are also regulated at the level of coactivating receptors [57]. Degranulation by NK cells following the triggering of NKG2D requires simultaneous engagement of co-activating receptors such as 2B4 or NTB-A by their ligands on target cells [57]. Therefore, the relatively low lytic activity induced by Vpr through ULBP-1 and -2 induction could be explained on the basis of lack of concomitant induction of coactivating ligands. Recently we demonstrated that co-activating ligands are important for NK cell destruction of HIV-1-infected cells [24] and that HIV-1 down modulates the coactivating receptor ligands [24],[25]. This would then make the infected cell less vulnerable to destruction since activated NK cells will have a reduced ability to degranulate. We are currently investigating how HIV-1 down modulates the ligands for the NK cell coactivating receptors in order to test this hypothesis.

Our observation that HIV-1 Vpr induces NK cell ligands opens a new set of questions that will need to be addressed in the near future. Specifically, it will be compelling to ascertain whether up-regulation of ULBP-1 and 2 by Vpr can be exploited therapeutically, such that HIV-1 infected cells can be manipulated to become more susceptible to NK lysis; (ii) whether other accessory or, in general, viral proteins can manipulate other aspects of the NK response; and (iii) the signaling steps linking activation of cell cycle checkpoint proteins and the increase in steady-state mRNA levels for NKG2D ligands.

## Materials and Methods

### Antibodies and fusion proteins

The mouse anti-human CD4, CD16, CD56, CD112 and MIC-A/MIC-B monoclonal antibodies (mAbs) were obtained from BD Biosciences (http://www. bdbiosciences.com/). The soluble fusion protein of the NKG2D receptor with the Fc portion of human IgG and mouse anti-human NKG2D, ULBP-1, ULBP-2, and -3 antibodies (Abs) were obtained from R&D Systems (www.rndsystems.com). The anti-MIC-A and MIC-B mAbs that do not cross-react with one another were obtained from Axxora (www.axxora.com). The fluorochrome-conjugated goat anti-human IgG1 Fc-specific secondary Ab (with minimal cross-species reaction) was obtained from Jackson ImmunoResearch Laboratories (www.jacksonimmuno.com) and the fluorochrome-conjugated rabbit anti-mouse IgG secondary Ab was from Dako (www.dako.com). The mouse anti-HIV-1 p24 mAb, KC57, was obtained from Beckman Coulter (Fullerton, CA) and the mouse monoclonal Ab clone AG3.0 was obtained from the National Institutes of Health AIDS Research and Reference Reagent Program (www.aidsreagent.org) and was deposited by Dr J. Allan [58].

### Cells and culture reagents

All primary cells used in this study were isolated from peripheral blood drawn from all healthy donors after informed written consent was obtained in accordance with the Declaration of Helsinki and the policies of the Institutional Review Board at Rush University Medical Center, Chicago, IL. Peripheral blood mononuclear cells (PBMC) were isolated by centrifugation (1000× *g*, 20 min 20°C of peripheral blood over a Ficoll-hypaque gradient (Mediatech, http://www.cellgro.com/). CD4^pos^ T-cells were isolated from PBMC by negative selection using a CD4^pos^ T-cell isolation kit (Dynal, http://www.invitrogen.com/) and stimulated with anti-CD3/anti-CD28 mAb-coated microbeads (Dynal) in RPMI complete medium that consisted of RPMI medium (Mediatech) supplemented with 10% heat inactivated (56°C, 30 min) fetal bovine serum (FBS) (Mediatech) and penicillin/streptomycin (Mediatech) before infection with DHIV or transduction with lentivirus vectors. The Jurkat E6-1 cell line was obtained from the American Type Culture Collection (http://www.atcc.org/) and was maintained in RPMI complete medium. The 293 FT cell line (Invitrogen), used in the generation of virus and vectors, was maintained according to the manufacturer's specifications. Caffeine was obtained from the Sigma Chemical Company (http://www.sigma.com/). KU55933 was obtained from Calbiochem (http://www.calbiochem.com). Aphidicolin was obtained from the Sigma Chemical Company. KU55933 and aphidicolin stock solutions were prepared by dissolving the drugs in DMSO (Sigma Chemical Company) at 1 mM concentration prior to their addition to medium at a final concentration of 10 µM.

### Virus vectors

The envelope-defective DHIV vector is isogenic to the HIV molecular clone HIV-1_NL4/3_. To construct DHIV vectors with premature stop-codons in the *nef*, *vpu*, *vif* and *vpr* open reading frames or containing mutated *vpr* genes, base changes were made by site-directed mutagenesis (Quikchange II XL, www.stratagene.com) of subcloned fragments and cloning the mutagenized fragments back into DHIV. All mutations were sequenced to verify accurate mutagenesis, and Western blotting of lysates from DHIV-infected CD4^pos^ Jurkat E6-1 cells was used to verify correct protein expression (Figure S8). DHIV-ΔVpu was verified to lack Vpu activity by its inability to down modulate CD4 molecules on primary T-cells (data not shown).

The FG12 vector system was graciously provided by Dr. Dong Sung An (University of California, Los Angles, CA). The DCAF1_3590 target sequences have been reported previously [42]. The scrambled sequence was 5′ GCATATCCACCGTGAGTGT 3′. All target sequences were obtained as olignucleotides (IDT, www.idtdna.com), annealed, and inserted downstream of the H1 RNA polymerase III promotor as described [59]. Western blots of lysates of primary activated T-cells transduced with the DCAF_3590 sequence produce less DCAF1 (Figure S9). pPR-Vip, the lentivirus vector that expressed HIV-1 Vpr and GFP, was generated as described previously [30].

Vesicular stomatitis virus (VSV)-G protein pseudotyped FG12, pPR-VIP and DHIV vectors were produced as previously described [36]. Viral vectors were titered by spin-inoculation at 1200×* g* for two hr at 20°C of dilutions of viral supernatant onto the Jurkat E6-1 cell line and detection of either intracellular HIV-1 p24 antigen (DHIV) or GFP expressing cells (FG12) by flow cytometry and the calculation of titers as described [36].

### Infections/transduction

Primary T-cells were activated using anti-CD3/anti-CD28 mAb coupled to magnetic beads for 48 hr for DHIV and pPR-VIP or 24 hr for the FG12 vector. Infection/transduction was done by spin-inoculated as described [60] at an MOI of five for DHIV, ten for the FG12 vector, and five for pPR-VIP. For HIV-1_NL4/3_ infection; an MOI of 0.01 was used. Following the infection, the cells were cultured in RPMI complete medium with 100 U/mL recombinant IL-2 (AIDS Research and Reference Reagent Program, Division of AIDS, NIAID, NIH, deposited by Dr. Maurice Gately, Hoffmann-La Roche Inc, Nutley, NJ; [61]). For experiments where cells were transduced with the FG12 vector and then infected with DHIV, CD4^pos^ T-cells were activated with anti-CD3/anti-CD28 mAb-coated microbeads, transduced with the FG12 vector, cultured for an additional 48 hr, and then infected with DHIV.

### Flow cytometry

Simultaneous detection of surface antigens and intracellular HIV-1 p24 antigen (Ag) was done as previously described [24]. Infected cells were designated by the absence of CD4 and the presence of HIV-1 p24 Ag (Figure S1). For analysis of cell cycle profiles, cells were stained for the appropriate surface markers, washed with FACS buffer (PBS containing 0.1% NaN_3_ and 2% FBS) fixed for 20 min on ice with 0.25% paraformaldehyde, and permeabilized for 20 min on ice with 0.1% Triton X-100 in PBS. Following a wash in FACS buffer, intracellular staining was performed for HIV-1 p24 Ag and washed a final time. DNA was stained using 1 µM TO-PRO-3 (Invitrogen) in FACS buffer supplemented with 11.25 Kunitz units/mL RNAse (Sigma) and promptly analyzed on an LSR II flow cytometer (BD).

### Purification of infected cells for cytotoxicity assays and real-time RT-PCR

Forty-eight hrs after infection culture, CD4^neg^ p24^pos^ cells were purified by removing CD4^pos^ p24^neg^ cells with anti-CD4 mAb coated magnetic beads (Dynal). To eliminate dead cells the Dead Cell removal kit from Miltenyi was used (http://www.miltenyibiotech.com/). Purity of infected cells was routinely greater than 95% as determined by flow cytometry.

### Real-time RT-PCR

Primer pairs for detection of ULBP-1, ULBP-2, ULBP-3, MIC-A and MIC-B were obtained from previous studies described in [62]. The primer pair used for GAPDH was 5′ GCACCGTCAAGGCTGAGAAC 3′ (sense) and 5′ GGATCTCGCTCCTGGAAGATG 3′ (antisense). Total RNA was isolated using the RNAqueous Kit (Ambion, www.ambion.com) and treated with DNAse (TURBO DNA-Free, Ambion). Reverse-transcription and real-time RT-PCR reactions were carried out using the Superscript III Platinum Two-Step qRT-PCR kit using Sybr Green chemistry (Invitrogen) according to the manufacturer's protocol. The cDNA was amplified in triplicate with the ABI 7500 Real-Time PCR System (Applied Biosystems, www.appliedbiosystems.com) with indicated primer pairs for 40 cycles at 95°C for 15 sec and 60°C for one min. Analysis was done by using the ΔC_T_ method for relative quantification as previously described [62] except that GAPDH was used as a reference. Similar amplification efficiencies for NKG2D ligand and GAPDH were demonstrated by analyzing serial cDNA dilutions with values of the slope of log cDNA amount vs. ΔC_T_ of <0.1. Threshold cycles (C_T_) for GAPDH (reference) and NKG2D ligands (sample) were determined in triplicate. We defined the values obtained for uninfected cells as standard values and determined the relative increase (rI) in copy numbers in relation to these standard values according to the formula: rI = 2̄−[(C_T_ Sample−C_T_ Reference)−(C_T_ Standard Sample−C_T_ Standard Reference)].

### Cytotoxicity assay

The cytotoxicity of HIV infected cells by autologous NK was measured using the ^51^Cr release assay. The cytotoxicity assay was done according to the methods previously described in [24].

## Supporting Information

Figure S1 Gating strategy used for detection of NKG2D ligands on infected cells. Infected primary T-cell blasts and uninfected CD4^pos^ T-cells were surface stained with anti-CD4 Ab. All cells were stained intracellularly for HIV-1 p24 antigen (Ag). Cells were then incubated in the presence of Aquadead stain kit (Invitrogen) to distinguish viable and non-viable cells. Throughout the study NKG2D ligands were evaluated on either 10^4^ viable uninfected (CD4^pos^ HIV-1 p24 Ag^neg^ cells) or 10^4^ viable infected cells (CD4^neg^ HIV-1 p24 Ag^pos^). FSC = forward scatter, SSC = side scatter. Gates in red indicate selection process for infected and uninfected cells.
(2.48 MB TIF)

Figure S2 NKG2D ligands are not expressed on CD4^pos^ T-cells infected with ΔVpr HIV-1. Infected primary T-cell blasts and uninfected CD4^pos^ T-cells were surface stained with a fusion protein of human NKG2D and the Fc portion of human IgG1 along with fluorochrome-conjugated goat anti-human IgG1. All cells were stained intracellularly for HIV-1 p24 antigen (Ag). Two-dimensional plots were derived following acquisition on a flow cytometer of 10^4^ viable cells. Markers in dot plots were positioned based on the staining controls. The figure is representative data from three separate experiments.
(0.98 MB TIF)

Figure S3 Effect of point mutations in postions 65 and 80 of Vpr on the cell cylcle of HIV-infected cells. Infected primary T-cell blasts and uninfected CD4^pos^ T-cells were stained with TO-PRO-3 in order to obtain the (G2+M)/G1 ratio.
(0.67 MB TIF)

Figure S4 Expression of DCAF1 is not affected by HIV-1 Vpr. The HeLa cell line was either treated with 10 µM aphidocolin (Aph), or infected with VSV-G pseudotyped HIV-1 with wild-type Vpr (Vpr) or HIV with Q65R and R80A mutations in Vpr (Vpr QR). Following treatment/infection cells lysates were made and western blotted. Western blots were probed with DCAF1 specific antibody.
(0.56 MB TIF)

Figure S5 Inhibition of ATR activity relieves Vpr-induced G_2_ arrest. Primary CD4^pos^ T-cell blasts were exposed to 4 mM of the ATR inhibitor, caffeine (B and D) or vehicle (A or C) and either infected with HIV-1 (C and D) or left uninfected (A and B). Forty-eight hrs. following exposure to caffeine and HIV-1 infection the cell cycle profile of the uninfected and infected cells were detected by TO-PRO-3 staining.
(1.50 MB TIF)

Figure S6 Inhibition of ATM activity reduces NKG2D ligand expression on primary CD4^pos^ T-cells treated with aphidocolin. Primary CD4^pos^ T-cells were treated with 10 µM aphidicolin in the presence of 10 µM KU55933 (ATM-specific inhibitor) or 4 mM caffeine. As a negative control aphidicolin-treated cells were exposed to vehicles used to dissolve the inhibitor in solution. Following 48 h exposure to KU55933, caffeine or vehicle the cells were stained with a fusion protein of human NKG2D and the Fc portion of human IgG1 and fluorochrome conjugated-goat anti-human IgG Fc specific antibody (blue line) or secondary antibody alone [staining control (red line)]. The histograms are gated for 10^4^ viable CD4^pos^ cells. This is a representative of two experiments.
(0.71 MB TIF)

Figure S7 Ability of NK cells to lyse autologous T-cells infected with HIV-1 lacking Vpr. Primary CD4^pos^ T-cell blasts were infected with HIV-1 that were deficient in expression of Vpr (ΔVpr). As a control, the same cells were infected with wild-type (WT) HIV-1. Following infection the infected cells were isolated, labeled with ^51^Cr and mixed with autologous NK cells at 2.5∶1 (A and C) and 5∶1 (B and D) effector cell to target cell ratios. Prior to the lytic assay some of the NK cells were exposed to blocking antibodies to NKG2D (C and D). At the end of the incubation period culture fluids were harvested and analyzed for the presence of ^51^Cr. Percent specific lysis was determined as described in the Materials and Methods section. Each point designates a sample from each group. Bars represent the mean percent specific lysis. This supplemental figure is a dot plot representation of Figure 10.
(1.02 MB TIF)

Figure S8 Expression of viral proteins by various HIV-1 mutants. HIV-1-infected CD4^pos^ cells were lysed in cell lysis buffer (1% NP-40, 150 mM NaCl, 50 mM Tris-HCl, pH 7.4, 0.25% Na-deoxycholate, 1 mM EDTA. 1 mM PMSF, 1 mM Na_3_VO_4_, 0.1% SDS, and protease inhibitors), run on 15% SDS-PAGE gels, transferred to PVDF, and probed for the indicated proteins with specific antibodies. (A) Lysates from CD4^pos^ cells infected with DHIVΔVpr, DHIVΔVif, DHIVΔVpr,ΔVif or DHIV containing Vpr with point mutations in specific residues. (B) Lyates from CD4^pos^ T-cells infected with DHIVΔNef.
(3.19 MB TIF)

Figure S9 Ability of shRNA expressing either scrambled or DCAF1 specific sequence to down modulate DCAF1 in transduced cells. Primary T-cell blasts were transduced with the shRNA with specific sequences, sorted by FACS to greater than 95% purity based on GFP expression, lysed in cell lysis buffer (1% NP-40, 150 mM NaCl, 50 mM Tris-HCl, pH 7.4, 0.25% Na-deoxycholate, 1 mM EDTA. 1 mM PMSF, 1 mM Na_3_VO_4_, 0.1% SDS, and protease inhibitors), run on 15% SDS-PAGE gels, transferred to PVDF, and probed for the either DCAF1 or β-actin proteins with specific antibodies.
(1.57 MB TIF)
