# Supplementary figures and images for: HIV-1 Vpr Triggers Natural Killer Cell–Mediated Lysis of Infected Cells through Activation of the ATR-Mediated DNA Damage Response
Source: PLoS Pathog. 2009 Oct 2;5(10):e1000613. doi: 10.1371/journal.ppat.1000613 (PMC2747015; doi:10.1371/journal.ppat.1000613)

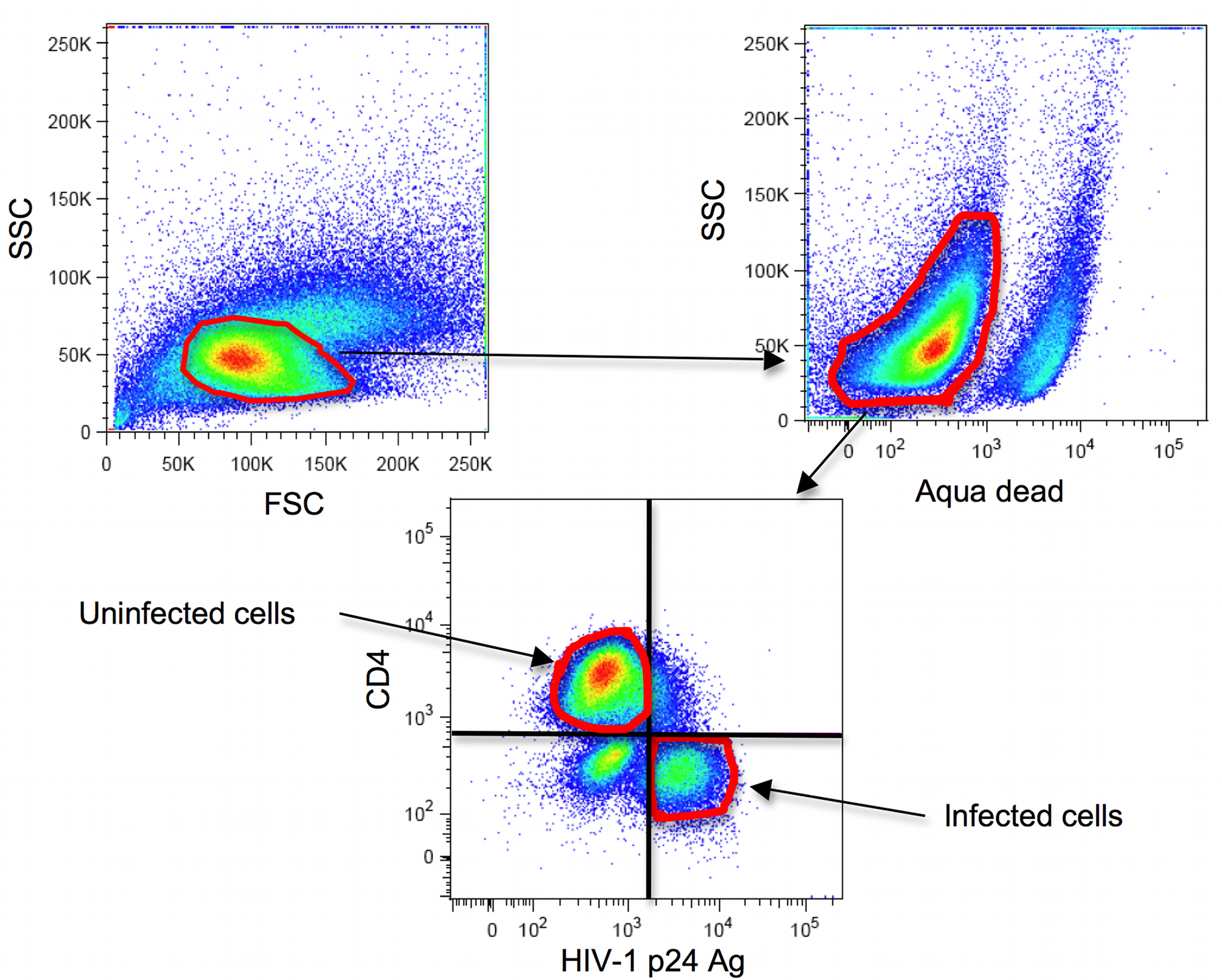

Supplement: Figure S1 — Gating strategy used for detection of NKG2D ligands on infected cells. Infected primary T-cell blasts and uninfected CD4pos T-cells were surface stained with anti-CD4 Ab. All cells were stained intracellularly for HIV-1 p24 antigen (Ag). Cells were then incubated in the presence of Aquadead stain kit (Invitrogen) to distinguish viable and non-viable cells. Throughout the study NKG2D ligands were evaluated on either 104 viable uninfected (CD4pos HIV-1 p24 Agneg cells) or 104 viable infected cells (CD4neg HIV-1 p24 Agpos). FSC = forward scatter, SSC = side scatter. Gates in red indicate selection process for infected and uninfected cells. (2.48 MB TIF) [file ppat.1000613.s001.tif]

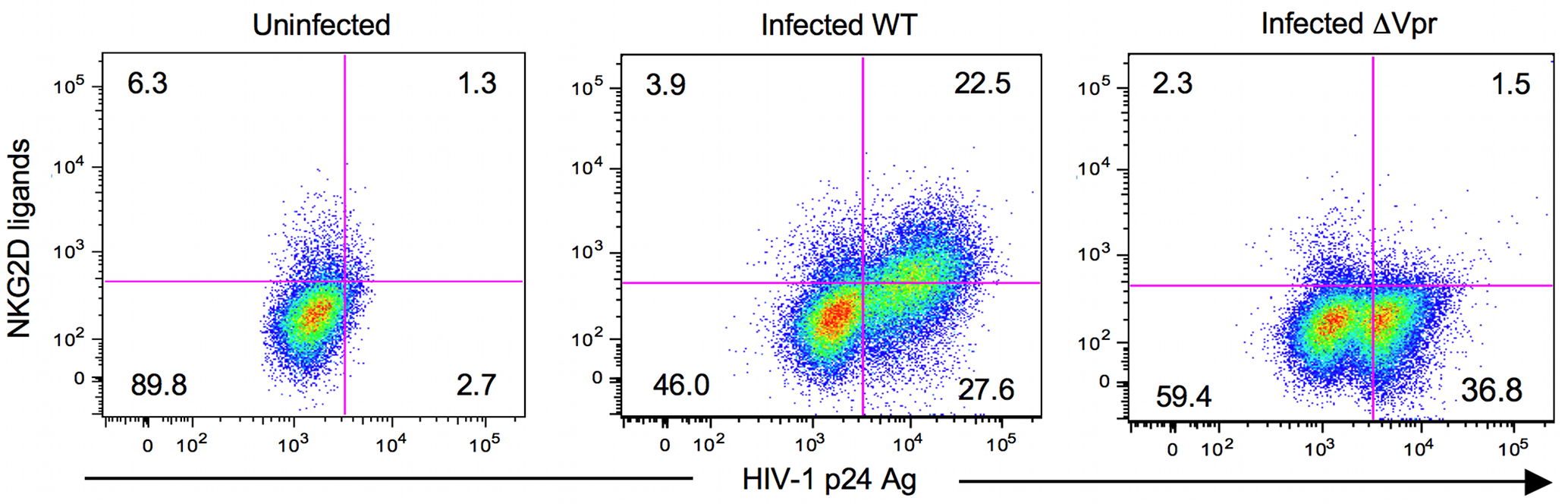

Supplement: Figure S2 — NKG2D ligands are not expressed on CD4pos T-cells infected with ΔVpr HIV-1. Infected primary T-cell blasts and uninfected CD4pos T-cells were surface stained with a fusion protein of human NKG2D and the Fc portion of human IgG1 along with fluorochrome-conjugated goat anti-human IgG1. All cells were stained intracellularly for HIV-1 p24 antigen (Ag). Two-dimensional plots were derived following acquisition on a flow cytometer of 104 viable cells. Markers in dot plots were positioned based on the staining controls. The figure is representative data from three separate experiments. (0.98 MB TIF) [file ppat.1000613.s002.tif]

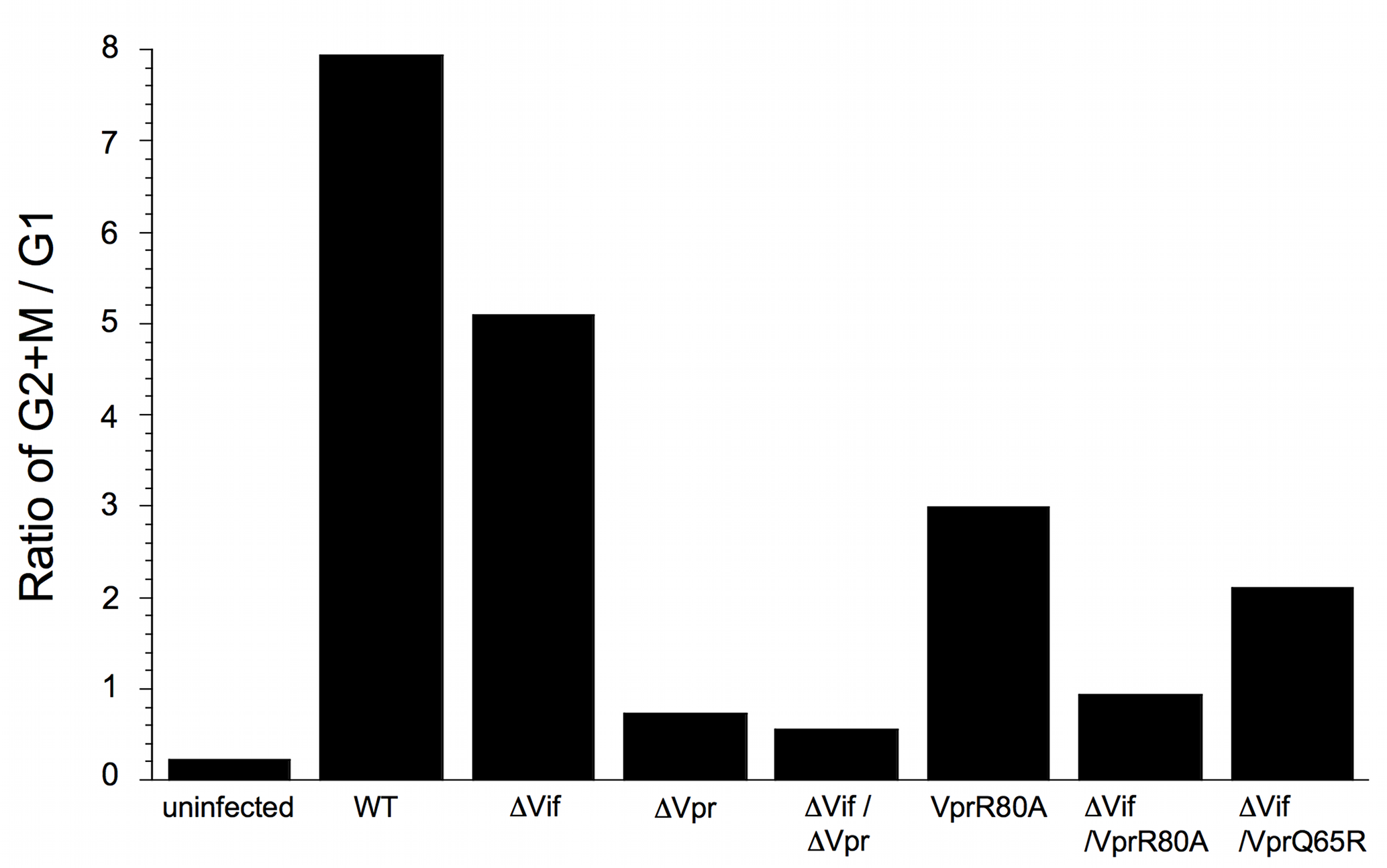

Supplement: Figure S3 — Effect of point mutations in postions 65 and 80 of Vpr on the cell cylcle of HIV-infected cells. Infected primary T-cell blasts and uninfected CD4pos T-cells were stained with TO-PRO-3 in order to obtain the (G2+M)/G1 ratio. (0.67 MB TIF) [file ppat.1000613.s003.tif]

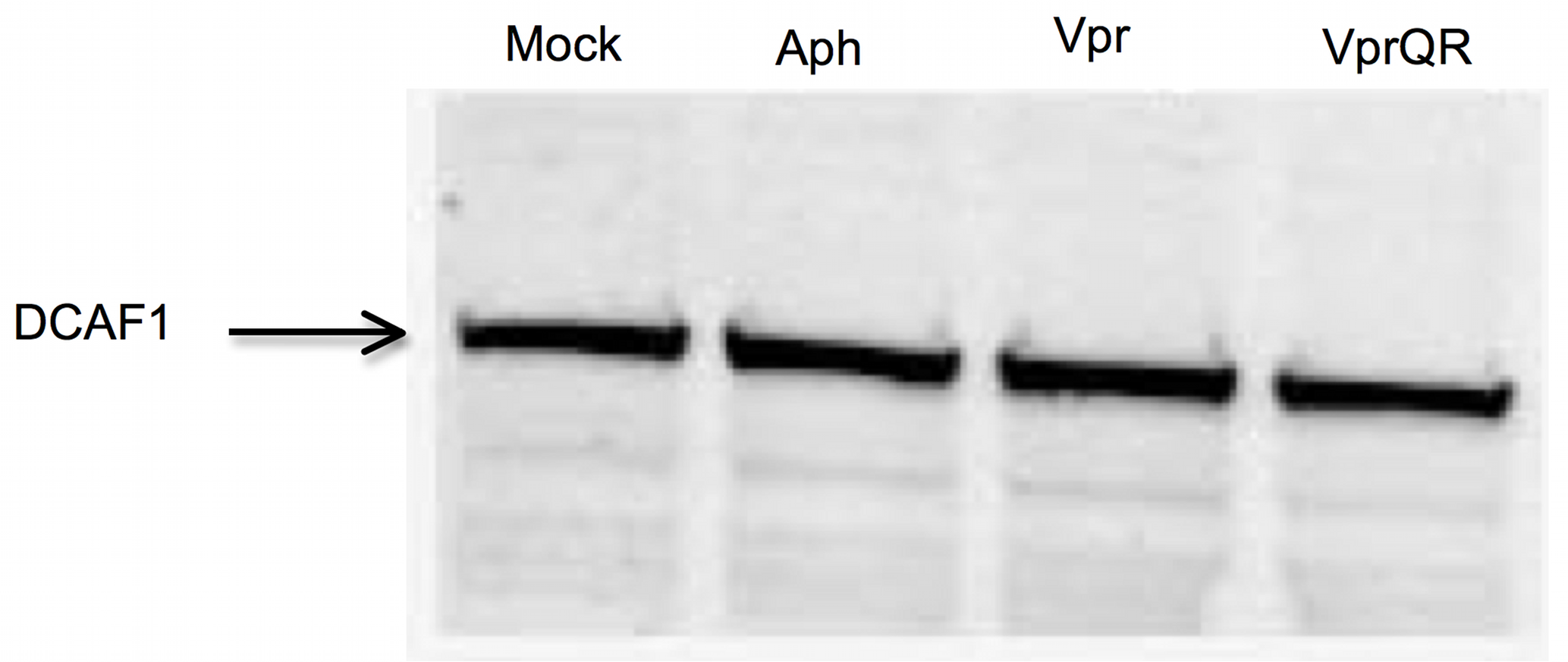

Supplement: Figure S4 — Expression of DCAF1 is not affected by HIV-1 Vpr. The HeLa cell line was either treated with 10 µM aphidocolin (Aph), or infected with VSV-G pseudotyped HIV-1 with wild-type Vpr (Vpr) or HIV with Q65R and R80A mutations in Vpr (Vpr QR). Following treatment/infection cells lysates were made and western blotted. Western blots were probed with DCAF1 specific antibody. (0.56 MB TIF) [file ppat.1000613.s004.tif]

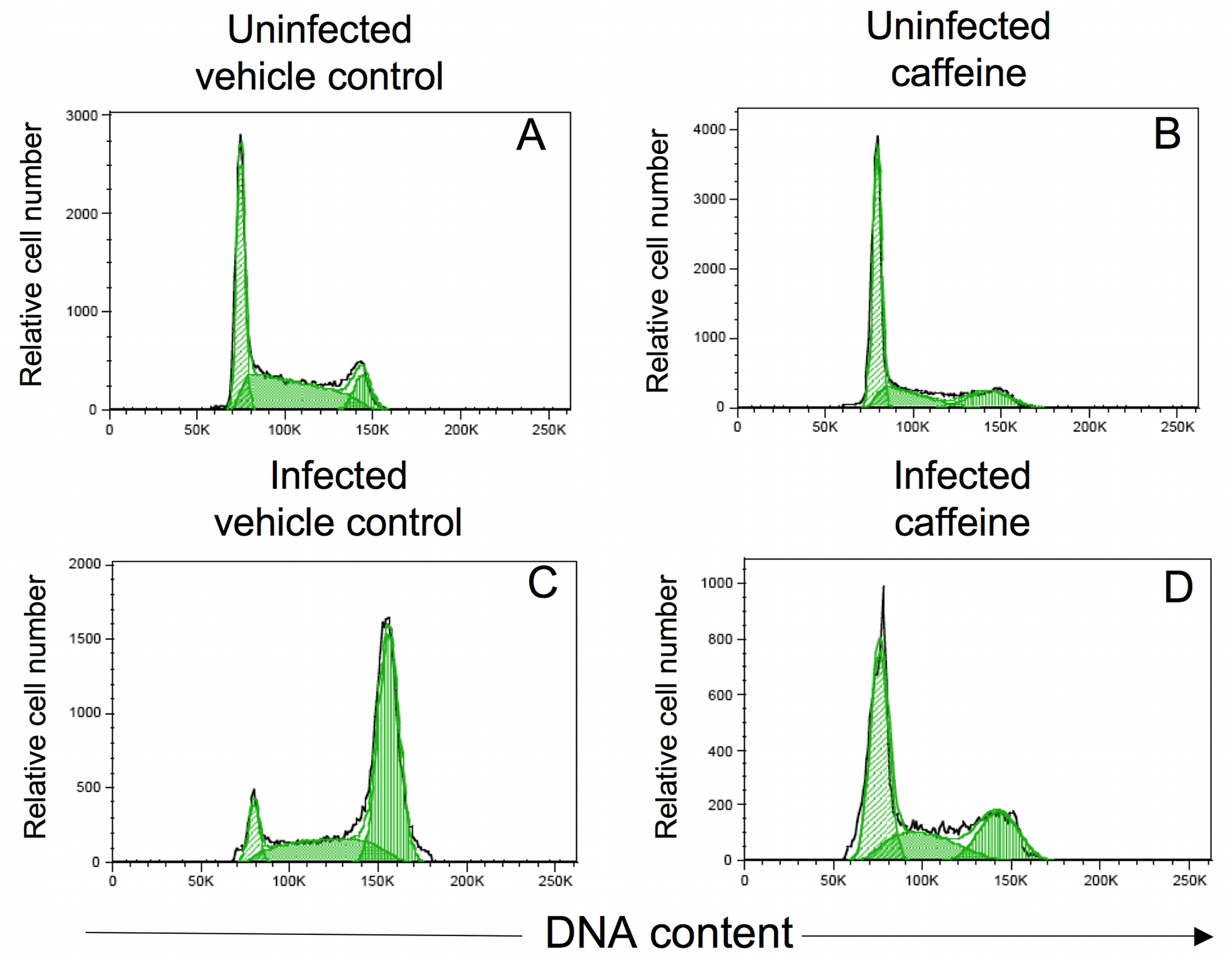

Supplement: Figure S5 — Inhibition of ATR activity relieves Vpr-induced G2 arrest. Primary CD4pos T-cell blasts were exposed to 4 mM of the ATR inhibitor, caffeine (B and D) or vehicle (A or C) and either infected with HIV-1 (C and D) or left uninfected (A and B). Forty-eight hrs. following exposure to caffeine and HIV-1 infection the cell cycle profile of the uninfected and infected cells were detected by TO-PRO-3 staining. (1.50 MB TIF) [file ppat.1000613.s005.tif]

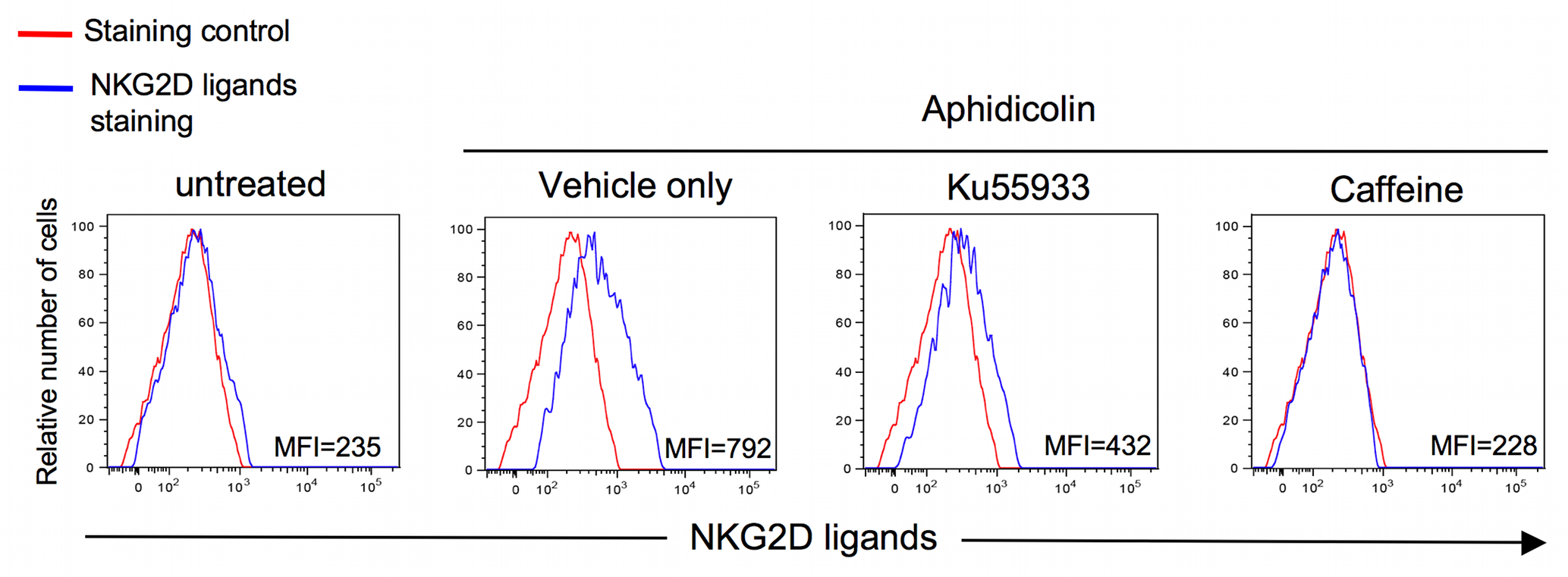

Supplement: Figure S6 — Inhibition of ATM activity reduces NKG2D ligand expression on primary CD4pos T-cells treated with aphidocolin. Primary CD4pos T-cells were treated with 10 µM aphidicolin in the presence of 10 µM KU55933 (ATM-specific inhibitor) or 4 mM caffeine. As a negative control aphidicolin-treated cells were exposed to vehicles used to dissolve the inhibitor in solution. Following 48 h exposure to KU55933, caffeine or vehicle the cells were stained with a fusion protein of human NKG2D and the Fc portion of human IgG1 and fluorochrome conjugated-goat anti-human IgG Fc specific antibody (blue line) or secondary antibody alone [staining control (red line)]. The histograms are gated for 104 viable CD4pos cells. This is a representative of two experiments. (0.71 MB TIF) [file ppat.1000613.s006.tif]

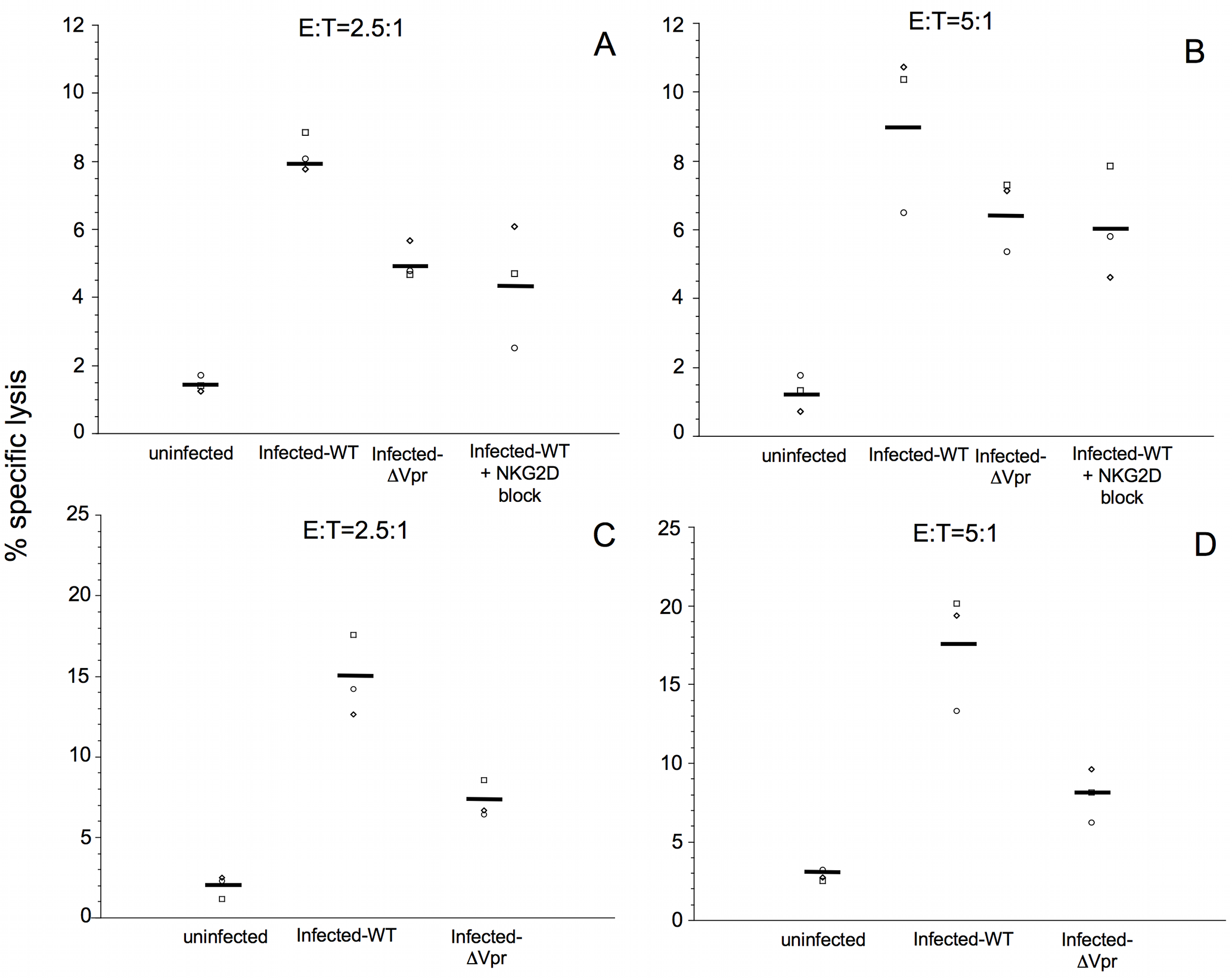

Supplement: Figure S7 — Ability of NK cells to lyse autologous T-cells infected with HIV-1 lacking Vpr. Primary CD4pos T-cell blasts were infected with HIV-1 that were deficient in expression of Vpr (ΔVpr). As a control, the same cells were infected with wild-type (WT) HIV-1. Following infection the infected cells were isolated, labeled with 51Cr and mixed with autologous NK cells at 2.5∶1 (A and C) and 5∶1 (B and D) effector cell to target cell ratios. Prior to the lytic assay some of the NK cells were exposed to blocking antibodies to NKG2D (C and D). At the end of the incubation period culture fluids were harvested and analyzed for the presence of 51Cr. Percent specific lysis was determined as described in the Materials and Methods section. Each point designates a sample from each group. Bars represent the mean percent specific lysis. This supplemental figure is a dot plot representation of Figure 10. (1.02 MB TIF) [file ppat.1000613.s007.tif]

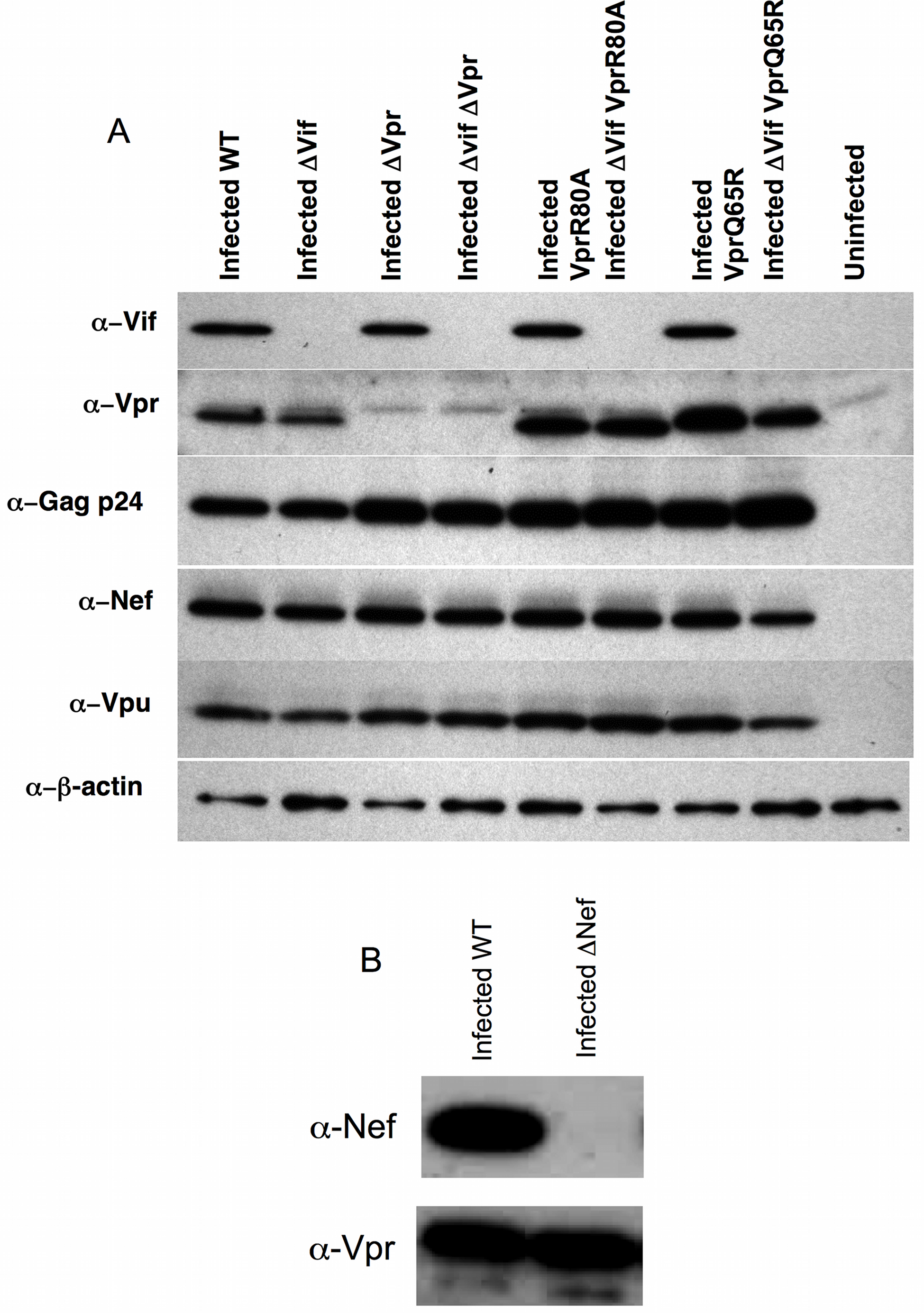

Supplement: Figure S8 — Expression of viral proteins by various HIV-1 mutants. HIV-1-infected CD4pos cells were lysed in cell lysis buffer (1% NP-40, 150 mM NaCl, 50 mM Tris-HCl, pH 7.4, 0.25% Na-deoxycholate, 1 mM EDTA. 1 mM PMSF, 1 mM Na3VO4, 0.1% SDS, and protease inhibitors), run on 15% SDS-PAGE gels, transferred to PVDF, and probed for the indicated proteins with specific antibodies. (A) Lysates from CD4pos cells infected with DHIVΔVpr, DHIVΔVif, DHIVΔVpr,ΔVif or DHIV containing Vpr with point mutations in specific residues. (B) Lyates from CD4pos T-cells infected with DHIVΔNef. (3.19 MB TIF) [file ppat.1000613.s008.tif]

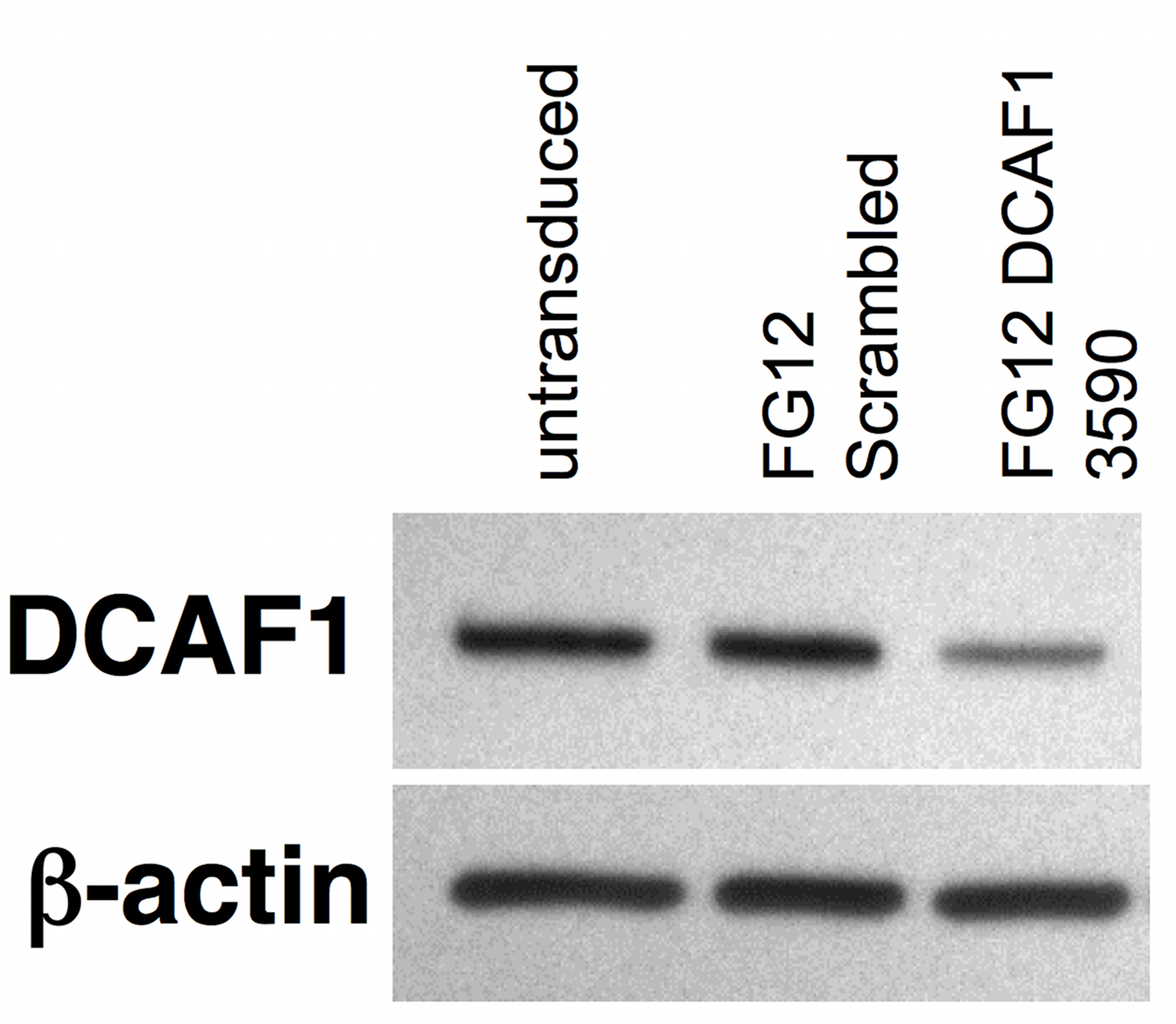

Supplement: Figure S9 — Ability of shRNA expressing either scrambled or DCAF1 specific sequence to down modulate DCAF1 in transduced cells. Primary T-cell blasts were transduced with the shRNA with specific sequences, sorted by FACS to greater than 95% purity based on GFP expression, lysed in cell lysis buffer (1% NP-40, 150 mM NaCl, 50 mM Tris-HCl, pH 7.4, 0.25% Na-deoxycholate, 1 mM EDTA. 1 mM PMSF, 1 mM Na3VO4, 0.1% SDS, and protease inhibitors), run on 15% SDS-PAGE gels, transferred to PVDF, and probed for the either DCAF1 or β-actin proteins with specific antibodies. (1.57 MB TIF) [file ppat.1000613.s009.tif]
